# A distinct gut microbiota composition in patients with ankylosing spondylitis is associated with increased levels of fecal calprotectin

**DOI:** 10.1186/s13075-019-2018-4

**Published:** 2019-11-27

**Authors:** Eva Klingberg, Maria K. Magnusson, Hans Strid, Anna Deminger, Arne Ståhl, Johanna Sundin, Magnus Simrén, Hans Carlsten, Lena Öhman, Helena Forsblad-d’Elia

**Affiliations:** 1 Department of Rheumatology and Inflammation Research, Sahlgrenska Academy at the University of Gothenburg, Gothenburg, Sweden; 2 Department of Rheumatology, Sahlgrenska University Hospital, Gröna stråket 14, SE-41345 Gothenburg, Sweden; 3 Department of Microbiology and Immunology, Sahlgrenska Academy at the University of Gothenburg, Gothenburg, Sweden; 4 Department of Internal Medicine, Södra Älvsborgs sjukhus, Borås, Sweden; 5 Department of Internal Medicine and Clinical Nutrition, Sahlgrenska Academy at the University of Gothenburg, Gothenburg, Sweden; 6 Center for Functional Gastrointestinal and Motility Disorders, University of North Carolina at Chapel Hill, Chapel Hill, NC USA; 7 Department of Public Health and Clinical Medicine, Rheumatology, Umeå University, Umeå, Sweden

**Keywords:** Ankylosing spondylitis, Spondyloarthritis, Microbiota, Intestinal inflammation, Inflammatory bowel disease

## Abstract

**Background:**

Ankylosing spondylitis (AS) shares many characteristics with inflammatory bowel disease (IBD). Intestinal microbiota most likely plays an important role in the development of IBDs and may also be involved in the pathogenesis of AS. We aimed to define and compare the fecal microbiota composition in patients with AS, ulcerative colitis (UC), and healthy controls (HC) and to determine relationships between fecal microbiota, fecal calprotectin, and disease-related variables in AS.

**Methods:**

Fecal microbiota composition was assessed with GA-map™ Dysbiosis Test (Genetic Analysis, Oslo, Norway), which also reports the degree of deviation of the microbiota composition compared with a healthy control population, a Dysbiosis Index (DI) score 1–5. The AS patients were assessed with questionnaires, back mobility tests, fecal calprotectin, erythrocyte sedimentation rate (ESR), and C-reactive protein (CRP).

**Results:**

Totally, 150 patients with AS (55% men, median age 55.5 years, median BASDAI 3.2), 18 patients with UC (56% men, median age 30.5 years), and 17 HC (65% men, median age 22 years) were included. Principal component analysis showed highly separate clustering of fecal microbiota from the patients with AS, UC, and HC. Compared with HC, fecal microbiota in AS was characterized by a higher abundance of *Proteobacteria*, *Enterobacteriaceae*, *Bacilli*, *Streptococcus* species, and *Actinobacteria*, but lower abundance of *Bacteroides* and *Lachnospiraceae*.

Further, fecal microbiota composition differed between patients with normal (≤ 50 mg/kg, *n* = 57) and increased (≥ 200 mg/kg, *n* = 36) fecal calprotectin. Patients with increased fecal calprotectin had lower abundance of bacteria with anti-inflammatory properties such as *Faecalibacterium prausnitzii* and *Clostridium* and higher abundance of the genus *Streptococcus*. No association was found between the fecal microbiota composition and HLAB27 status, disease activity, function, or medication. Dysbiosis (defined as DI ≥ 3) was found in 87% of AS patients.

**Conclusions:**

Patients with AS have a distinct fecal microbiota signature, which is linked to fecal calprotectin levels, a marker of intestinal inflammation, but not to other clinical parameters. These findings suggest a local interplay between intestinal microbiota and gut inflammation in AS.

**Trial registration:**

ClinicalTrials.gov, NCT00858819. Registered March 9, 2009.

## Background

Ankylosing spondylitis (AS) is a chronic inflammatory disease that shares several clinical, pathogenetic, and pathophysiologic characteristics with the inflammatory bowel diseases (IBD), ulcerative colitis (UC), and Crohn’s disease (CD). Besides chronic inflammation of the spine, sacroiliac joints, entheses, and peripheral joints, AS is characterized by microscopic intestinal inflammation, which has been demonstrated in 40–60% of the patients [1–3]. AS patients also have an increased risk of developing IBD, especially CD [4–6]. The histopathology of the chronic form of intestinal inflammation in AS resembles CD, with presence of granulomas, activation of Paneth cells, and increased production of anti-microbial peptides [7–9]. Interleukin (IL) 23 and IL17, which are key cytokines in AS, are produced in the inflamed gut, both in AS and in IBD [10]. Active intestinal inflammation has been associated with increased disease activity in AS, more pronounced bone marrow edema of the sacroiliac joints in non-radiographic axial spondyloarthritis (nr-axSpA), and higher risk of development of AS from nr-axSpA [2, 5, 6, 11, 12]. This indicates a link between the inflammation in the gut and the locomotor system.

The gastrointestinal tract is the home of more than 1000 species of bacteria, but also fungi and viruses, which coexist with the host in a reciprocal relationship. The gut microbiota is necessary for the development and shaping of the immune system, and the host genetics play a role in the establishing and shaping of the gut microbiota [13]. Intestinal microbiota most likely play a role in initiating and triggering the immune system in individuals who are genetically susceptible for IBD, leading to the typical gut inflammation of CD and UC [14]. Aberrations in the gut microbiome, dysbiosis with decreased bacterial diversity, expansion of potentially pro-inflammatory bacteria, and reduction of potentially anti-inflammatory, protective bacteria have repeatedly been shown in IBD [15–17]. However, it is still unclear whether the dysbiosis in IBD is a cause or a consequence of the gut inflammation.

In a cohort of AS patients followed for 5 years, we have previously shown that two thirds of the patients had elevated fecal calprotectin levels, which was predictive of the development of CD [6]. The aims of the present study were to evaluate differences in fecal microbiota composition between patients with AS, patients with UC, and healthy controls. Further, we aimed to determine potential relationships between fecal microbiota composition, intestinal inflammation measured indirectly by fecal calprotectin, and disease-related variables in the AS patients.

## Methods

### Subjects of the study

#### Patients with AS

Patients with a diagnosis of AS according to the modified New York criteria were recruited from three rheumatology clinics in the west of Sweden [18]. Exclusion criteria were psoriasis, diagnosis of IBD, pregnancy, and difficulties in understanding Swedish language. All patients fulfilling the study criteria were invited to participate. In total, 204 patients were included in 2009, and the same patients were invited to a 5-year follow-up in 2014. The current study is based on data from the 5-year follow-up. At the 5-year follow-up, all patients were assessed by the same physician (AD) for swollen and tender joints count and back mobility. Back mobility, disease activity, and function were assessed with the Bath Ankylosing Spondylitis Metrology Index (BASMI), Ankylosing Spondylitis Disease Activity Score (ASDAS_CRP_), Bath Ankylosing Spondylitis Disease Activity Index (BASDAI), Bath Ankylosing Spondylitis patient Global score (BAS-G), and the Bath Ankylosing Spondylitis Functional Index (BASFI) [19]. Blood samples were analyzed for hemoglobin, erythrocyte sedimentation rate (ESR), and C-reactive protein (CRP) using standard laboratory techniques, and the patients were asked to send in a stool sample. The presence of the HLAB27 antigen was assessed by HLA typing with sequence-specific oligonucleotide primers (PCR-SSO) by LABType® (One Lambda, Inc., CA, USA) and use of the Luminex platform. In total, 150 patients provided a stool sample at the 5-year follow-up with enough material to be used for microbiota and fecal calprotectin analyses.

#### Patients with UC

Eighteen treatment-naïve patients with newly diagnosed UC were recruited from Sahlgrenska University Hospital (Gothenburg) and Södra Älvsborgs Hospital (Borås). The UC diagnosis was based on endoscopic and histological findings. The patients had not received any antibiotics during the month before inclusion. The disease activity of the patients with UC was evaluated using the Mayo score, which contains four variables: stool frequency, rectal bleeding, endoscopic findings, and the physician’s global assessment. Each variable is graded from 0 to 3, and the maximum total score is 12 [20]. The extent of disease was classified into proctitis, left-sided colitis, or extensive colitis (beyond the left colonic flexure) according to the Montreal classification [21].

#### Healthy controls

Seventeen healthy controls with no prior history of gastrointestinal or other chronic disorders were recruited at Sahlgrenska University Hospital (Gothenburg). None of the healthy controls had any gastrointestinal complaints during the last week prior to inclusion, assessed using a standardized questionnaire. Further, none of the healthy controls had taken any immunosuppressive agents, antibiotics, or any other medication during the last 3 months prior inclusion.

### Ethical approval

All patients and healthy controls in the study gave their written informed consent. The research protocol was approved by the Regional Ethics Committee in Gothenburg and carried out in accordance with the Helsinki Declaration.

### Stool samples and fecal calprotectin

Stool samples were collected and sent in to the laboratory by the patients and healthy controls. The samples were immediately frozen and stored at − 20 °C.

The stool samples were analyzed for fecal calprotectin using an enzyme-linked immunosorbent assay (ELISA) kit (Bühlmann Laboratories AG, Schönenbuch, Switzerland). Calprotectin, which is a cytosolic protein abundant in neutrophils and belonging to the calcium-binding calgranulins or S100 proteins, is a marker of intestinal inflammation, but its concentration in feces is also increased by, for example, the use of non-steroidal anti-inflammatory drugs (NSAIDs) [22]. A fecal calprotectin ≤ 50 mg/kg was defined as normal, and a value ≥ 200 mg/kg was defined as increased. The threshold 200 mg/kg was chosen since it was considered a reasonable level on which to initiate further endoscopic investigation in a patient [23].

### Analysis of fecal microbiota

Microbiota analysis of fecal samples from the patients with AS, patients with UC, and healthy controls was performed using the GA-map™ Dysbiosis Test (Genetic Analysis, Oslo, Norway), which consists of 54 DNA probes targeting ≥ 300 bacteria on different taxonomic levels. The probes have been selected based on the ability to distinguish between healthy controls, irritable bowel syndrome (IBS), and IBD patients [24]. The results are given as abundances of bacteria denoted as probe signal intensity (PSI). The test also algorithmically assesses fecal bacterial abundance and profile in comparison with a healthy reference group at the laboratory. A deviation in the microbiome from normobiosis is summarized in a Dysbiosis Index (DI) score (1–5). DI ≥ 3 indicates a microbiota that differs from the healthy reference group. The bacterial profile used to create the DI score is based on 15 different bacteria (defined by Genetic Analysis AS): *Ruminococcus albus/bromii*, *Ruminococcus gnavus*, *Faecalibacterium prausnitzii*, *Lactobacillus*, *Streptococcus sanguinis* and *Streptococcus salivarius thermophilus*, *Dialister invisus*, *Akkermansia muciniphila*, *Bacteroides fragilis*, *Alistipes*, *Shigella*/*Escherichia*, *Bifidobacterium*, *Bacteroides*/*Prevotella*, *Firmicutes* (*Bacilli*), *Firmicutes* (*Clostridia*), and *Proteobacteria*. The normobiotic reference in the DI was based on fecal samples collected from 165 healthy donors in Sweden and Norway, with no clinical signs or symptoms of gut disorder [24]. The GA-map™ Dysbiosis Test has been used in studies on IBD, IBS, scleroderma, Sjogren’s syndrome, and obesity, but never before in AS, to the best of our knowledge [25–29].

### Statistical analyses

Statistical analyses were made using SPSS Statistics version 25 (IBM, Chicago, USA). Descriptive statistics are presented as median and interquartile range (IQR). In comparisons between two groups, the Mann-Whitney *U* test was used for continuous variables and the chi-square test or Fisher’s exact test for categorical variables. Correlations were calculated using Spearman’s correlation (*r*_s_). All tests were two-tailed. A Bonferroni corrected *p* value of < 0.0009 was considered statistically significant.

Multivariate factor analysis (SIMCA-P+ software; Umetrics, Umeå, Sweden version 15) was used to examine the relationship between categorical variables (*Y*-variables) and detection levels of bacteria (*X*-variables). The microbiota composition in the patients with AS, patients with UC, and healthy controls was analyzed with principal component analysis (PCA). Orthogonal partial least squares discriminant analyses (OPLS-DA) were used to correlate a selected *Y*-variable and multiple *X*-variables with each other in linear multivariate models to further investigate the differences between groups and to determine which variables had the largest discriminatory power. The following *Y*-variables were explored with OPLS-DA: (1) patients with AS compared with healthy controls, (2) AS patients with normal (≤ 50 mg/kg, *n* = 57) vs increased (≥ 200 mg/kg, *n* = 36) fecal calprotectin, (3) HLAB27 positive (84.7%, *n* = 127) vs. negative (15.3%, *n* = 23) AS patients, and (4) dichotomized levels (below vs. above median value and first vs. fourth quartile) of indices of disease activity, back mobility, and function in the AS patients, i.e., BASDAI, ASDAS-CRP, BASMI, BASFI, CRP, and ESR.

The quality of the OPLS-DA was based on the parameters *R*2, i.e., the goodness of fit of the model (values of ≥ 0.5 define good discrimination, best possible fit, R2 = 1), and Q2, i.e., the goodness of prediction of the model (values of ≥ 0.5 or no more than 0.3 lower than the R2 value, define predictive ability). To reduce the risk of overfitting, CV-ANOVA tests and post hoc 100 permutation tests of OPLS-DA models were performed. Models with *p* < 0.05 and permutation indices fulfilling the post hoc analysis criteria of intercepts of R2Y ≤ 0.4 and Q2Y < 0.05 were accepted [30].

## Results

### Clinical characteristics of the patients

The characteristics of the 150 patients with AS, 18 patients with UC, and 17 healthy controls are demonstrated in Table 1. Notably, in contrast to the patients with AS, the healthy controls and patients with UC were younger and not taking any NSAID or immunosuppressant. There was also a discrepancy in disease duration between the patients with AS and UC.

**Table 1:** The characteristics of the patients with ankylosing spondylitis (AS), ulcerative colitis (UC), and healthy controls (HC)

	AS (*n* = 150)	UC (*n* = 18)	HC (*n* = 17)
Women/men, *n* (%)	68 (45.3)/82 (54.7)	8 (44.4)/10 (55.6)	6 (35.5)/11 (64.7)
Age, years	55.5 (46–67)	30.5 (27–39)	22 (21–31)
AS symptom duration, years	28.5 (18–39)		
HLAB27 positive, *n* (%)	127 (84.7)		
BAS-G, score	2.8 (1.5–5.9)		
ASDAS-CRP, score	2.1 (1.3–1.7)		
BASDAI, score	3.2 (1.8–5.2)		
BASFI, score	2.3 (1.1–4.1)		
BASMI, score	3.4 (2.4–4.6)		
ESR, mm/h	8 (4–14)		
CRP, mg/L	3 (1–6)	5 (3.5–16.5)	1 (1–1)
Fecal calprotectin, mg/kg	80 (0–190)	606 (29–10,320)	All < 15
Patients on NSAIDs, *n* (%)	115 (76.7)	0	0
- Daily use of NSAIDs	61 (40.7)		
- On-demand use of NSAIDs	54 (36.0)		
On TNFi all, *n* (%)	35 (23.3)	0	N.A.
- TNFi in monotherapy	16 (10.7)		
- TNFi + methotrexate	19 (12.7)		
On DMARD monotherapy	16 (10.7)	0	N.A.
- Methotrexate	9 (6.0)		
- Sulfasalazine	7 (4.7)		

Data is presented as median (interquartile range) or number (%)

*ASDAS-CRP* Ankylosing Spondylitis Disease Activity Score based on CRP, *BASDAI* Bath Ankylosing Spondylitis Disease Activity Index, *BAS-G* Bath Ankylosing Spondylitis patient Global score, *BASFI* Bath Ankylosing Spondylitis Functional Index, *BASMI* Bath Ankylosing Spondylitis Metrology Index, *CRP* C-reactive protein, *DMARD* disease modifying anti-rheumatic drug, *ESR* erythrocyte sedimentation rate, *NSAID* non-steroidal anti-inflammatory drug, *TNFi* tumor necrosis factor inhibitor

Among the patients with UC, the median total Mayo score was 7 (IQR 5.7–8.3). Three (16.7%) patients presented with proctitis, 3 (16.7%) patients presented with left-sided colitis, and 12 (66.7%) suffered from extensive colitis.

### The microbiota composition in patients with AS compared with patients with UC and healthy controls

**Fig. 1:**
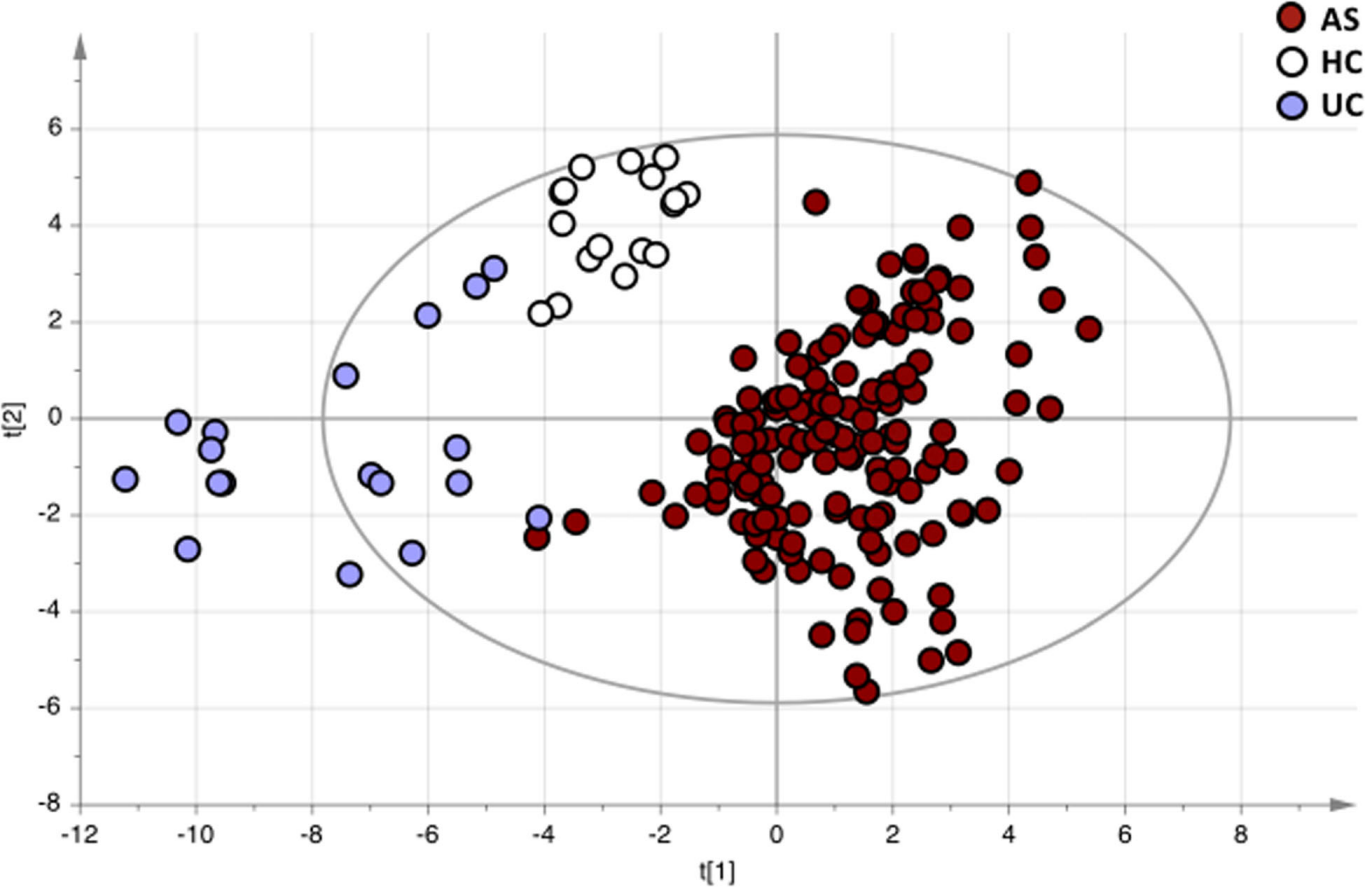
Fecal microbiota composition in patients with ankylosing spondylitis (AS, n=150), patients with ulcerative colitis (UC, *n* = 18), and healthy controls (HC, *n* = 17) analyzed by the GA-map™ Dysbiosis Test. Total variance of the analyzed 54 bacterial targets are shown in the principal component analysis (PCA)

Based on the fecal microbiota composition, patients with AS, patients with UC, and healthy controls clustered separately in a PCA, indicating major differences in the microbiota profile between the groups (Fig. 1).

**Fig. 2:**
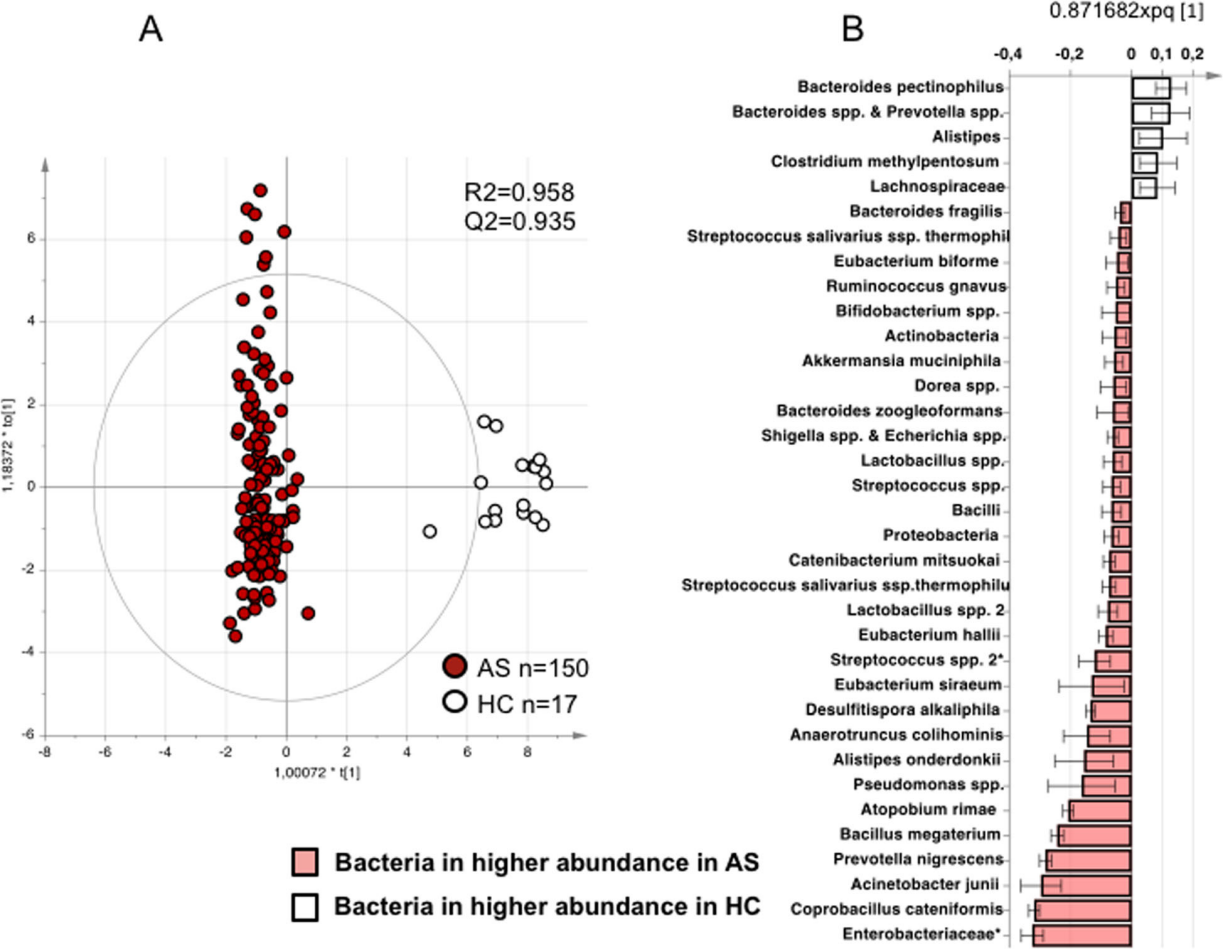
Comparison of fecal microbiota composition in patients with ankylosing spondylitis (AS) and healthy controls (HC). Fecal samples were analyzed by the GA-map™ Dysbiosis Test and evaluated using orthogonal partial least squares discriminant analysis (OPLS-DA). AS (*n* = 150) and HC (*n* = 17). **a** Score scatter plot from the OPLS-DA showing the separation between patients with AS (red circles) and HC (white circles). R2 defines the goodness of fit, and Q2 the goodness of prediction. **b** Loading column plot from the OPLS-DA. White columns represent bacteria in higher abundance in HC, and red columns represent bacteria in higher abundance in AS patients. All bacteria were included in the analysis, but the loading column plot shows only bacteria which abundance differed between the groups. Error bars represent 95% confidence interval. All analyzed bacteria are listed in Additional file 1: Table S1

To further define the differences in microbiota composition between AS and healthy controls, an OPLS-DA was performed, which showed excellent discrimination and predictive ability (R2 = 0.958, Q2 = 0.935, *p* < 0.0001) (Fig. 2a, b). Permutation test showed that the model was well fitted (intercepts: R2Y = 0.205 and Q2Y = − 0.388). In comparison with healthy controls, the fecal microbiota in AS was characterized by a higher abundance of *Proteobacteria*, *Enterobacteriaceae*, *Bacilli*, *Streptococcus* species, and *Actinobacteria*, but lower abundance of *Bacteroides* and *Lachnospiraceae.* A complete list of the bacteria is shown in Additional file 1: Table S1.

### Fecal microbiota composition and association to fecal calprotectin in the AS patients

The fecal microbiota composition of the AS patients with normal fecal calprotectin (≤ 50 g/kg, *n* = 57) was compared to patients with increased fecal calprotectin (≥ 200 mg/kg, *n* = 36).

**Fig. 3:**
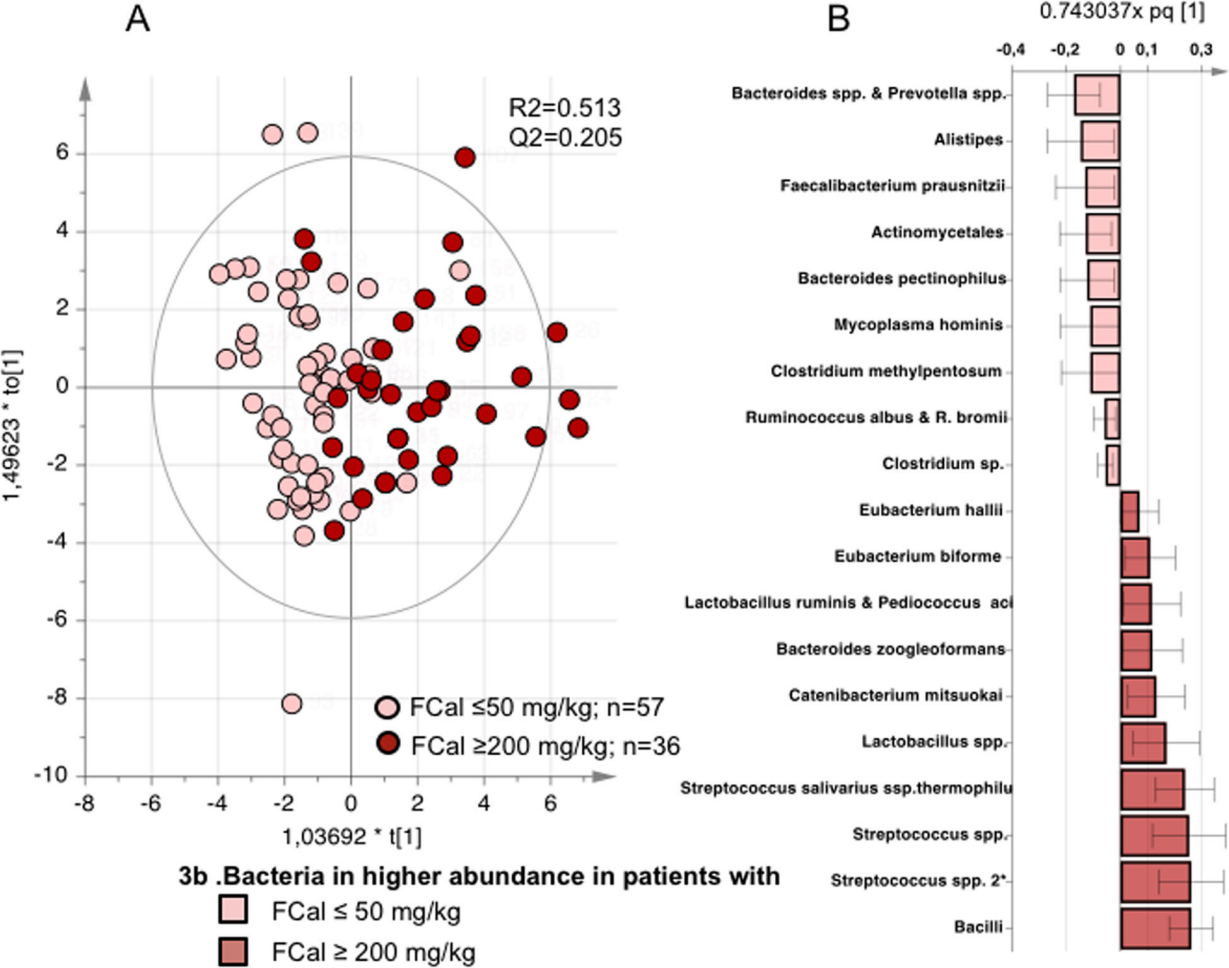
Fecal microbiota composition in relation to levels of fecal calprotectin. Comparison of the fecal microbiota composition in ankylosing spondylitis (AS) patients with normal (≤ 50 mg/kg, *n* = 57) vs. increased (≥ 200 mg/kg, *n* = 36) fecal calprotectin (Fcal). Orthogonal partial least squares discriminant analysis (OPLS-DA) was used to define fecal microbial differences between the groups. **a** Score scatter plot from the OPLS-DA showing the separation between patients with normal Fcal (pink circles) and increased Fcal (red circles). R2 defines the goodness of fit, and Q2 the goodness of prediction. **b** Loading column plot from the OPLS-DA. Pink columns represent bacteria in higher abundance in AS patients with normal fecal, and red columns represent bacteria in higher abundance in AS patients with increased Fcal. All bacteria were included in the analysis, but the loading column plot shows only bacteria which abundance differed between the groups. Error bars represent 95% confidence interval. All analyzed bacteria are listed in Additional file 1: Table S2

An OPLS-DA demonstrated that the fecal microbiota composition of patients with normal fecal calprotectin levels discriminated from patients with increased fecal calprotectin levels, although the predictive ability was modest (R2 = 0.513, Q2 = 0.205, *p* = 0.0004) (Fig. 3a, b). Permutation test showed that the model was well fitted (intercepts: R2Y = 0.307, Q2Y = − 0.331).

The AS patients with normal fecal calprotectin levels had higher abundance of *Bacteroides*, *Clostridium*, *Prevotella*, *Actinomycetales*, and *Faecalibacterium prausnitzii*, whereas patients with increased fecal calprotectin levels had a higher abundance of *Bacilli* class, *Streptococcus* genus, and *Lactobacillus* genus. A complete list of the bacteria which differed between patients with normal respective increased calprotectin is shown in Additional file 1: Table S2. Correlations between fecal calprotectin and bacteria in all the AS patients (*n* = 150) are shown in Additional file 1: Table S3, and scatterplots between calprotectin and bacteria in users and non-users of NSAIDs are shown in Additional file 2: Figure S1.

Fecal calprotectin was associated with several clinical parameters. Fecal calprotectin was higher in NSAID users compared with non-users (median (IQR) 88 (43–220) vs. 36 (19–120) mg/kg; *p* = 0.005) and weakly positively correlated with ASDAS-CRP (*r*_S_ = 0.191, *p* = 0.019), CRP (*r*_S_ = 0.252, *p* = 0.002), and BASMI (*r*_S_ = 0.203, *p* = 0.013). No association was found between fecal calprotectin and reported gastrointestinal symptoms.

### Fecal microbiota in relation to HLAB27, disease activity, function, and medication in the AS patients

Weak correlations (Spearman’s rho) were found between PSI values for a few bacteria and measures of disease activity (Additional file 1: Table S3). None of these correlations however reached a *p* value of < 0.0009, which was the threshold for statistical significance after the Bonferroni correction. Further, there was no significant difference (*p* value < 0.0009) in the PSI value of any bacteria between users and non-users of NSAIDs, TNFi or csDMARDs, or between smokers and non-smokers. No association was found between fecal microbiota composition and reported gastrointestinal symptoms.

Multivariate analysis of the microbiota composition using OPLS-DA could not discriminate between HLAB27 positive or negative patients or between patients with dichotomized levels (below vs. above median value and first vs. fourth quartile) of parameters reflecting disease activity or function (BASDAI, ASDAS-CRP, BASMI, BASFI, CRP, and ESR) (Additional file 1 Table S4).

### Dysbiosis Index score in patients with AS, patients with UC, and healthy controls

**Fig. 4:**
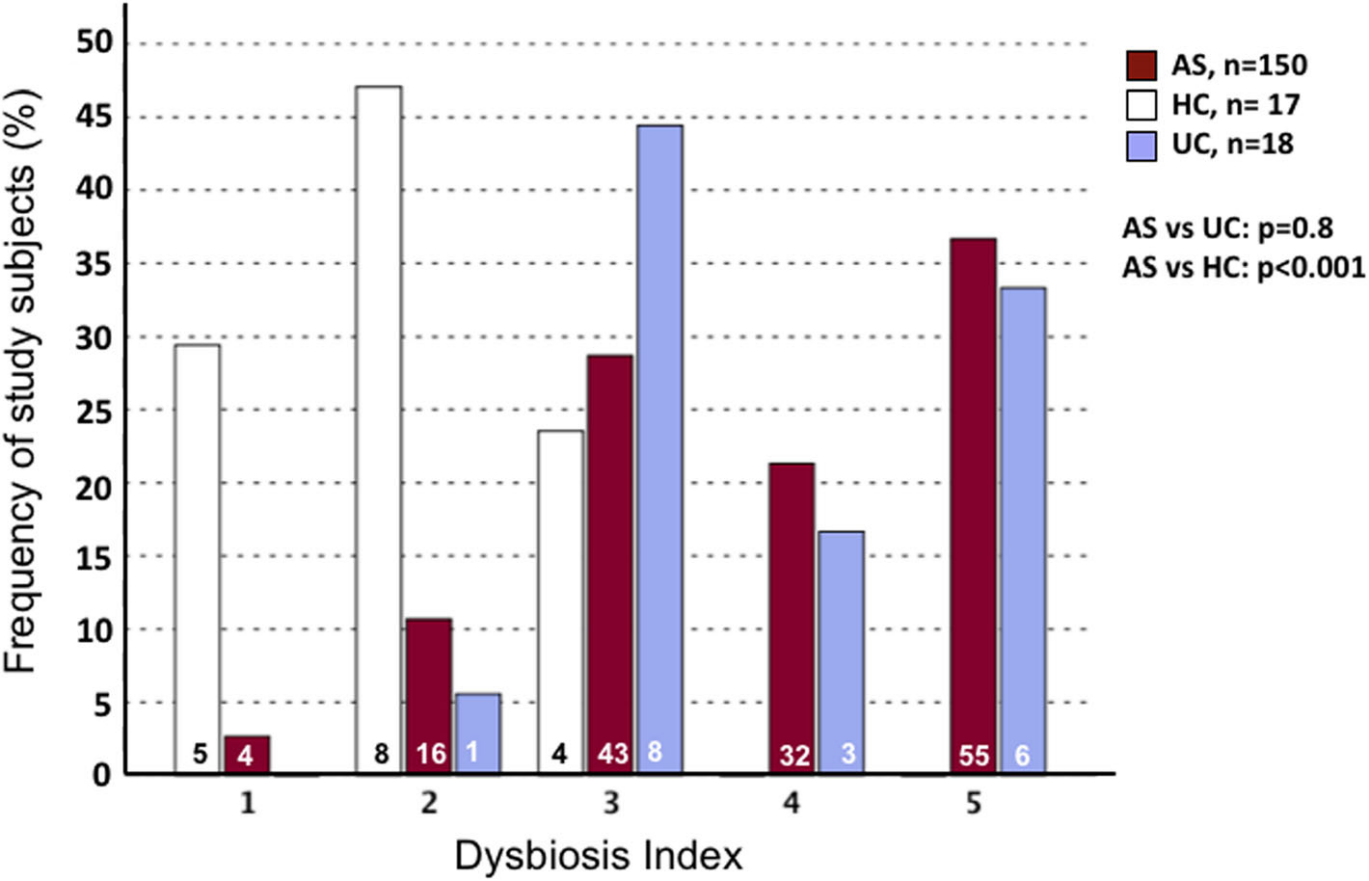
The distribution of the Dysbiosis Index (DI) score among patients with ankylosing spondylitis (AS, *n* = 150), patients with ulcerative colitis (UC, *n* = 18), and healthy controls (HC, *n* = 17). Fecal samples were analyzed by the GA-map™ Dysbiosis Test. DI is scored between 1 and 5, where a score of 1 and 2 signifies normobiosis and 3–5 dysbiosis of increasing severity. Digits inside the columns represent the number of subjects within each column

Fecal microbial dysbiosis, defined as a Dysbiosis Index (DI) score ≥ 3, was found in 131 (86.7%) of the patients with AS, in 17 (94.4%) of the patients with UC, and in 4 (23.5%) of the healthy controls [24]. The distribution of the DI score among the AS patients was similar to that of the UC patients (*p* = 0.8) but differed significantly from that of the healthy controls (*p* < 0.001) (Fig. 4).

**Fig. 5:**
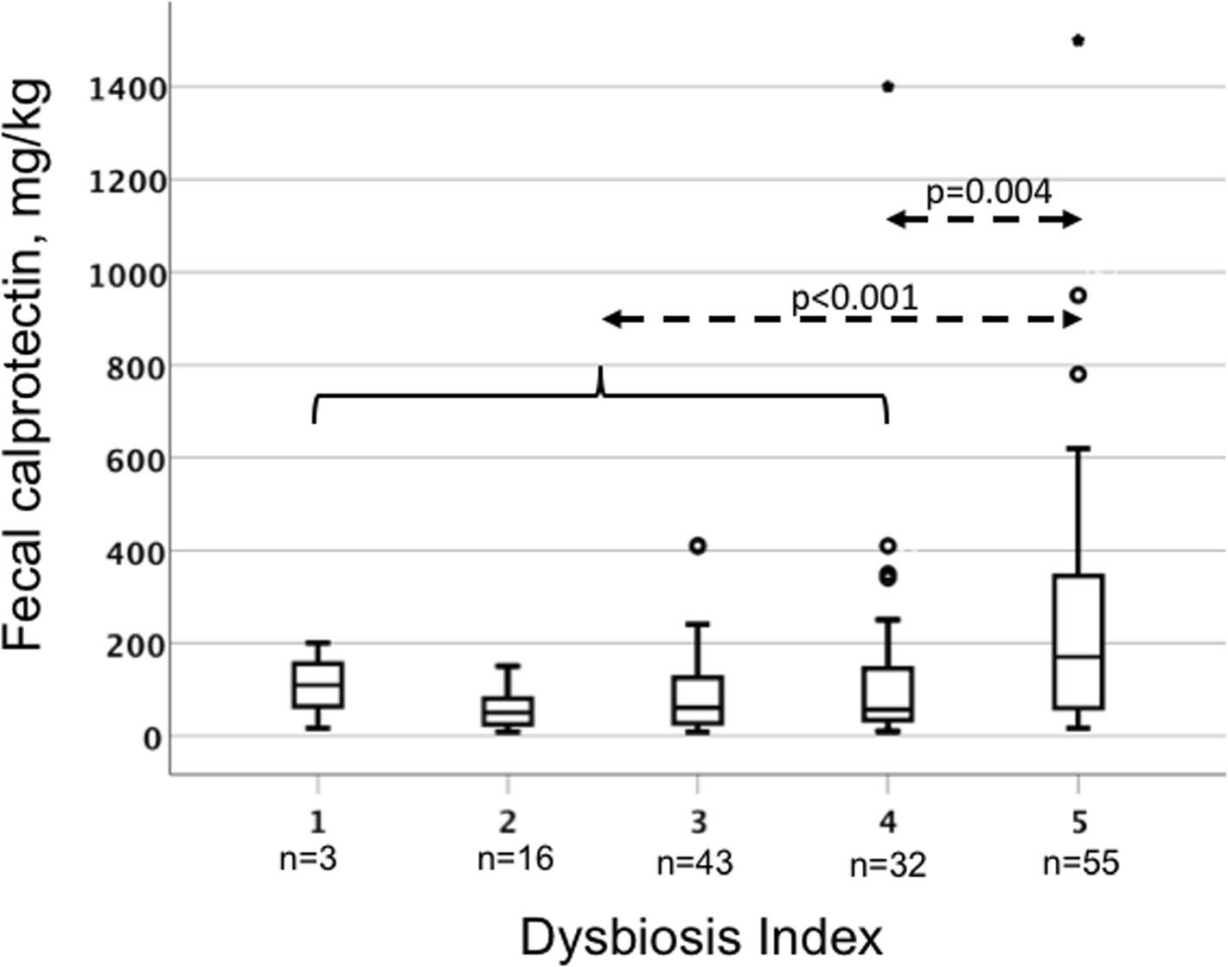
Fecal calprotectin (Fcal) in relation to dysbiosis index (DI) in patients with ankylosing spondylitis (AS). Boxplots represent the Fcal concentration within each DI score. Values are the medians (horizontal line), interquartile range (box), and range (whiskers). Outliers: circles show cases with values between 1.5 and 3.0 box lengths and stars (extremes) values more than 3 box lengths from the upper or lower edge of the box. Fcal was compared between groups of patients with different DI score using the Mann-Whitney *U* test. AS patients with the most pronounced dysbiosis (DI = 5) had significantly higher Fcal than patients with a DI of 4 (*p* = 0.004) and patients with a DI 1–4 (*p* < 0.001)

The AS patients with the most pronounced dysbiosis, DI = 5 (36.7%), had significantly higher fecal calprotectin than the patients with DI < 5 (63.3%) (170 (58–360) mg/kg vs. 58 (27–120) mg/kg; *p* < 0.001 (Fig. 5). DI was also positively correlated with fecal calprotectin (*r*_S_ = 0.303; *p* < 0.001). No association was found between DI and medication. Boxplots of the DI among users and non-users of NSAIDs and TNFi are shown in Additional file 3: Figure S2 and Additional file 4: Figure S3.

## Discussion

We studied the fecal microbiota composition in patients with AS, patients with UC, and healthy controls and found evidence for a distinct fecal microbiota signature in AS, which differed significantly from the patients with UC and healthy controls in the study. The fecal microbiota composition of the AS patients showed association with fecal calprotectin, but not with other clinical parameters. Thus, no clear association was found between the overall fecal microbiota composition and HLAB27 status, disease activity, physical function, medication, or smoking status. Dysbiosis was found in 88% of the AS patients, and an increased dysbiosis was associated with elevation of fecal calprotectin.

Several of our findings indicate that there are similarities in the aberrations of the gut microbiota in IBD and AS. We found higher abundance of the phylum *Proteobacteria*, especially the family *Enterobacteriaceae* and the genus *Shigella* and *Escherichia* among the AS patients compared with healthy controls. *Proteobacteria* is a phylum, consisting of Gram-negative staining bacteria containing pro-inflammatory lipopolysaccharides (LPS) in their cell membrane, which is overrepresented in the gut in several conditions characterized by chronic inflammation [31]. Similar to the findings of the present study, *Enterobacteriaceae*, belonging to the *Gammaproteobacteria*, have repeatedly been found to be enriched in the gut in UC and CD [17, 32–34]. Adherent-invasive *Escherichia coli* (AIEC), belonging to the family of *Enterobacteriaceae*, which can persist and replicate inside epithelial cells and macrophages are increased in the ileal mucosa in CD [35–37]. The presence of adherent and invasive bacteria, mainly *Escherichia coli* and *Prevotella*, has also been reported in AS in association with gut inflammation and damage of the intestinal mucosal barrier [38]. An increase in the *Gammaproteobacteria Erwinia* and *Pseudomonas* and a decrease in *Lachnospiraceae* have also been shown in reactive arthritis [39].

The AS patients with an elevated fecal calprotectin (≥ 200 mg/kg) had a relative decrease in the genus *Clostridium* and the species *Faecalibacterium prausnitzii* and *Bacteroidetes*. Both *F. prausnitzii* and *Clostridium* have been shown to have immune-suppressive effects [40]. Decreased levels of *Clostridiales* and *F.prausnitzii* have been found in CD and UC, and low abundance of the bacteria is associated with higher recurrence of CD after surgery and poorer effect of treatment with infliximab in CD and UC [28, 32, 40, 41]. *F.prausnitzii* produces the short-chain fatty acid (SCFA) butyrate, an important nutrient for epithelial cells. The bacterium has been found to have immune-suppressive effects on peripheral blood mononuclear cells in vitro, to produce a protein which inhibits the NF-κВ pathway, to stimulate the production of IL-10 and to be able to inhibit experimental colitis in BALB/c mice [40, 42]. An earlier study on the fecal microbiota in children with enthesitis-related arthritis reported findings similar to ours with lower abundance of *F. Prausnitzii* and the family *Lachnospiraceae* among the patients [43]. The AS patients with a fecal calprotectin ≥ 200 mg/kg in the current study had an increase of the genus *Streptococcus*. Interestingly, a gain in *Streptococcus* in stool samples has also been found in new-onset CD and has been associated with higher recurrence of CD after surgery [32, 41, 44]. Thus, several of the bacteria which we found to be increased or decreased respectively in AS have previously been reported to be increased and decreased in studies on IBD, with an extra strong resemblance with early CD. The findings suggest that similar microbial mechanisms may be involved in the pathogenesis of gut inflammation in the diseases and give further food for thought that subclinical gut inflammation in AS could be viewed as a preclinical CD. Yet, the fecal microbiota of the AS patients differed greatly from the UC patients in the current study, which may be explained by the much more inflamed state of the gut mucosa of the UC patients.

A large proportion (77%) of the AS patients of this study were using NSAIDs, and intestinal bacteria play a role in NSAID enteropathy [45, 46]. Further, NSAID use may alter the gut microbiota composition [47, 48]. In the present study, the microbiota composition did however not discriminate between users and non-users of NSAIDs.

There are earlier studies on the gut microbiota in AS or axial SpA, which have all found significant differences in the fecal microbiota composition in AS or SpA compared with healthy controls [49–52]. Tito et al. examined ileal and colonic biopsies in patients with newly diagnosed AS or nr-axSpA in relation to gut histology and found differences in the microbiota composition between patients with or without microscopic gut inflammation [50]. The study also reported a positive correlation between the abundance of the genus *Dialister* and ASDAS and BASDAI. Breban et al. studied the microbiota in fecal samples from patients with SpA, rheumatoid arthritis, and healthy controls and reported an increased abundance of the species *Ruminococcus gnavus* in SpA, especially in SpA patients with a history of IBD, and a positive correlation between *Ruminococcus gnavus* and BASDAI [51]. The current study confirms the findings of a distinct microbiota composition in AS and supports the prior report of differences in the microbiota between AS patients with or without subclinical gut inflammation. We also found that the abundance of *Ruminococcus gnavus* was higher in AS than in healthy controls. Conversely, we found no associations between the fecal microbiota composition and disease activity. There are differences between the studies regarding the methods used for microbiota analyses and sampling niche, mucosal biopsies vs. feces, which may have affected the results. Active gut inflammation has been associated with increased disease activity in AS [2, 5, 6, 12]. Our results indicate that there may be an interaction between intestinal bacteria and inflammation in the gut in AS, but we found no evidence for a direct link between the intestinal microbiota composition and other AS-related disease activity measures.

Strengths of the present study were the well-characterized cohorts and a large number of patients with AS. Limitations of the study were that the microbiota analysis was based on a defined set of bacterial probes instead of metagenomic sequencing and that the patients were assessed with fecal calprotectin, but not with endoscopy. A major limitation of the study was also the discrepancy between the patients with AS, UC, and healthy controls in regard to age, number of participants, medication, and disease duration, which may have affected the results. The study also lacked a control group with CD.

## Conclusions

We have demonstrated a distinct fecal microbiota signature in the patients with AS, which differed from the patients with UC and healthy controls. In the AS patients, fecal microbiota signature was linked to fecal calprotectin levels, a marker of intestinal inflammation, but not to other clinical parameters. This suggests that the intestinal microbiota may be involved in an interplay with subclinical gut inflammation in AS.

## Supplementary information

**Additional file 1: Table S1.** Comparison of the bacterial composition in patients with ankylosing spondylitis (AS, *n* = 150) and healthy controls (HC, *n* = 17). **Table S2.** Comparison of the bacterial composition in patients with ankylosing spondylitis (AS) with normal (≤ 50 mg/kg) versus increased (≥200 mg/kg) fecal calprotectin. **Table S3.** Correlations (Spearman’s Rho) between Probe Signal Intensity (PSI) of fecal bacteria and fecal calprotectin and parameters reflecting disease activity and function in 150 patients with ankylosing spondylitis. All correlations with a *p*-value ≤0.05 are shown. A Bonferroni corrected p-value of < 0.0009 was considered statistically significant (marked with *). **Table S4.** Comparison of the fecal microbiota composition in groups of ankylosing spondylitis patients with dichotomized levels (below vs. above median value and first vs. fourth quartile) of indices of disease activity, back mobility and function. Comparisons were also made between users vs. non-users of medication and smokers vs. non-smokers. Orthogonal partial least squares discriminant analysis (OPLS-DA) was used to define fecal microbial differences between the groups. The quality of OPLS-DA was based on the parameters R2, i.e., the goodness of fit of the model (values of ≥0.5 define good discrimination, best possible fit, R2 = 1), and Q2, i.e., the goodness of prediction of the model (values of ≥0.5 define high predictive ability).

**Additional file 2: Figure S1.** Scatterplots of Probe Signal Intensity (PSI) of fecal bacteria (Y-axis) and fecal calprotectin (mg/kg) (X-axis) in AS patients. Patients on NSAIDs are represented by circles, patients not on NSAIDs represented by squares.

**Additional file 3: Figure S2.** Boxplot showing the distribution of the Dysbiosis Index among non-users, on-demand users and daily users of non-steroidal anti-inflammatory drugs (NSAIDs).

**Additional file 4: Figure S3.** Boxplot showing the distribution of the Dysbiosis Index among non-users and users of tumour necrosis factor inhibitors (TNFi).

## Data Availability

The datasets analyzed during the current study are available from the corresponding author on reasonable request.

## Abbreviations

AIEC: Adherent-invasive *Escherichia coli*
AS: Ankylosing spondylitis
ASDAS-CRP: Ankylosing Spondylitis Disease Activity Score based on CRP
BASDAI: Bath Ankylosing Spondylitis Disease Activity Index
BASFI: Bath Ankylosing Spondylitis Functional Index
BAS-G: Bath Ankylosing Spondylitis patient Global score
BASMI: Bath Ankylosing Spondylitis Metrology Index
CD: Crohn’s diseases
CRP: C-reactive protein
cs/bDMARDs: Conventional synthetic or biologic disease modifying anti-rheumatic drugs
DI: Dysbiosis Index
DMARD: Disease modifying anti-rheumatic drug
ELISA: Enzyme-linked immunosorbent assay
ESR: Erythrocyte sedimentation rate
IBD: Inflammatory bowel disease
IBS: Irritable bowel syndrome
IL: Interleukin
IQR: Interquartile range
LPS: Lipopolysaccharides
nr-axSpA: Non-radiographic axial spondyloarthritis
NSAID: Non-steroidal anti-inflammatory drug
OPLS-DA: Orthogonal partial least squares discriminant analyses
PCA: Principal component analysis
PSI: Probe signal intensity
SpA: Spondyloarthritis
TNFi: Tumor necrosis factor inhibitor
UC: Ulcerative colitis
