# Supplementary material for: A distinct gut microbiota composition in patients with ankylosing spondylitis is associated with increased levels of fecal calprotectin
Source: Arthritis Res Ther. 2019 Nov 27;21:248. doi: 10.1186/s13075-019-2018-4 (PMC6880506; doi:10.1186/s13075-019-2018-4)
Supplement: Supplementary file 1 — Additional file 1: Table S1. Comparison of the bacterial composition in patients with ankylosing spondylitis (AS, n = 150) and healthy controls (HC, n = 17). Table S2. Comparison of the bacterial composition in patients with ankylosing spondylitis (AS) with normal (≤ 50 mg/kg) versus increased (≥200 mg/kg) fecal calprotectin. Table S3. Correlations (Spearman’s Rho) between Probe Signal Intensity (PSI) of fecal bacteria and fecal calprotectin and parameters reflecting disease activity and function in 150 patients with ankylosing spondylitis. All correlations with a p-value ≤0.05 are shown. A Bonferroni corrected p-value of < 0.0009 was considered statistically significant (marked with *). Table S4. Comparison of the fecal microbiota composition in groups of ankylosing spondylitis patients with dichotomized levels (below vs. above median value and first vs. fourth quartile) of indices of disease activity, back mobility and function. Comparisons were also made between users vs. non-users of medication and smokers vs. non-smokers. Orthogonal partial least squares discriminant analysis (OPLS-DA) was used to define fecal microbial differences between the groups. The quality of OPLS-DA was based on the parameters R2, i.e., the goodness of fit of the model (values of ≥0.5 define good discrimination, best possible fit, R2 = 1), and Q2, i.e., the goodness of prediction of the model (values of ≥0.5 define high predictive ability). [file 13075_2019_2018_MOESM1_ESM.docx]

**Addtional file 1: Table S1**

Comparison of the bacterial composition in patients with ankylosing spondylitis (AS, n=150) and healthy controls (HC, n=17)

| **A) Bacteria in higher abundance in AS than in HC**  **Phylum**, c-class, o-order, f-family, g-genus*, s-species* | **B) Bacteria in higher abundance in HC than in AS**  **Phylum**, c-class, o-order, f-family, g-genus*, s-species* |
| --- | --- |
| **Proteobacteria**, f-Enterobacteriaceae | **Bacteroidetes**, *s-Bacteroides pectinophilus* |
| **Firmicutes**, *s-Coprobacillus cateniformis* | **Bacteroidetes**, g-Bacteroides spp. & g-Prevotella spp. |
| **Proteobacteria**, *s-Acinetobacter junii* | **Bacteroidetes**, g-Alistipes |
| **Bacteroidetes**, *s-Prevotella nigrescens* | **Firmicutes**, *s-Clostridium methylpentosum* |
| **Firmicutes**, *s-Bacillus megaterium* | **Firmicutes**, f-Lachnospiraceae |
| **Actinobacteria**, *s-Atopobium rimae* |  |
| **Proteobacteria**, g-Pseudomonas spp |  |
| **Bacteroidetes**, *s-Alistipes onderdonkii* |  |
| **Firmicutes**, *s-Anaerotruncus colihominis* |  |
| **Firmicutes**, *s-Desulfitispora alkaliphila* |  |
| **Firmicutes**, *s-Eubacterium siraeum* |  |
| **Firmicutes**, g-Streptococcus spp. 2*** |  |
| **Firmicutes**, s-*Eubacterium hallii* |  |
| **Firmicutes**, g-Lactobacillus spp. 2 |  |
| **Firmicutes**, *s-Streptococcus salivarius ssp.thermophilus* |  |
| **Firmicutes,** *s-Catenibacterium mitsuokai* |  |
| **Proteobacteria** |  |
| **Firmicutes**, c-Bacilli |  |
| **Firmicutes**, g-Streptococcus spp |  |
| **Firmicutes**, g-Lactobacillus spp. |  |
| **Proteobacteria**, g-Shigella spp. & g-Echerichia spp. |  |
| **Bacteroidetes**, *s-Bacteroides zoogleoformans* |  |
| **Firmicutes**, g-Dorea spp |  |
| **Verrucomicrobia**, *s-Akkermansia muciniphila* |  |
| **Actinobacteria**, c-Actinobacteria |  |
| **Actinobacteria**, g-Bifidobacterium sp. |  |
| **Firmicutes**, *s-Ruminococcus gnavus* |  |
| **Firmicutes***, s-Eubacterium biforme* |  |
| **Firmicutes**, *s-Streptococcus salivarius ssp.thermophilus and S.Sanguinis* |  |
| **Bacteroidetes**, *s-Bacteroides fragilis* |  |

| **C) Bacteria in similar abundance in AS and HC**  **Phylum**, c-class, o-order, f-family, g-genus*, s-species* |
| --- |
| **Actinobacteria**, o-Actinomycetales |
| **Bacteroidetes**, g-Bacteroides spp. |
| **Bacteroidetes**, *s-Bacteroides stercoris* |
| **Firmicutes**, c-Clostridia |
| **Firmicutes**, *s-Dialister invisus* |
| **Firmicutes**, *s-Dialister invisus & s-Megasphaera micronuciformis* |
| **Firmicutes**, *s-Eubacterium rectale* |
| **Firmicutes**, *s- Faecalibacterium prausnitzii* |
| **Firmicutes** (various)* |
| **Firmicutes*** |
| **Firmicutes**, *s-Lactobacillus ruminis & s-Pediococcus acidilactici* |
| **Tenericutes**, *s-Mycoplasma hominis* |
| **Bacteroidetes**, *s-Parabacteroides johnsonii* |
| **Bacteroidetes**, g-Parabacteroides spp. |
| **Firmicutes**, g-Phascolarctobacterium sp. |
| **Firmicutes**, *s-Ruminococcus albus & s-R. bromii* |
| **Firmicutes**, *s-Streptococcus agalactiae and s-Eubacterium rectale* |
| **Firmicutes**, g-Veillonella spp. |

**Additional file 1: Table S2**

Comparison of the bacterial composition in patients with ankylosing spondylitis (AS) with normal (≤ 50 mg/kg) versus increased (≥200 mg/kg) fecal calprotectin

| **A) Bacteria in higher abundance in AS patients with fecal calprotectin ≤ 50 mg/kg**  **Phylum,** c-class, o-order, f-family, g-genus*, s-species* | **B) Bacteria in higher abundance in AS patients with fecal calprotectin ≥ 200 mg/kg**  ***Phylum,*** *c-class, o-order, f-family, g-genus, s-species* |
| --- | --- |
| **Bacteroidetes**, g-Bacteroides spp. & g-Prevotella spp. | **Firmicutes**, c-Bacilli |
| **Bacteroidetes**, g-Alistipes | **Firmicutes**, g-Streptococcus spp.2 |
| **Firmicutes**, *s-Faecalibacterium prausnitzii* | **Firmicutes**, *s*-*Streptococcus spp.* |
| **Actinobacteria**, c-Actinomycetales | **Firmicutes**, *s*-*Streptococcus salivarius ssp.thermophilus* |
| **Bacteroidetes**, *s*-*Bacteroides pectinophilus* | **Firmicutes**, g-Lactobacillus spp. |
| **Tenericutes**, *s-Mycoplasma hominis* | **Firmicutes**, *s*-*Catenibacterium mitsuokai* |
| **Firmicutes**, *s-Clostridium methylpentosum* | **Bacteroidetes**, *s*-*Bacteroides zoogleoformans* |
| **Firmicutes**, *s*-*Ruminococcus albus & s*-*R. bromii* | **Firmicutes**, *s*-*Lactobacillus ruminis & s*-*Pediococcus acidilactici* |
| **Firmicutes**, g-Clostridium spp. | **Firmicutes**, *s*-*Eubacterium Biforme* |
|  | **Firmicutes**, s-*Eubacterium hallii* |

| **C) Bacteria in similar abundance in AS patients with fecal calprotectin ≤ 50 and ≥ 200 mg/kg**  **Phylum,** c-class, o-order, f-family, g-genus*, s-species* |
| --- |
| **Proteobacteria,** *s-Acinetobacter junii* |
| **Actinobacteria** |
| **Verrucomicrobia,** *s-Akkermansia muciniphila* |
| **Bacteriodetes,** *s-Alistipes onderdonkii* |
| **Firmicutes,** *s-Anaerotruncus colihominis* |
| **Actinobacteria,** *s-Atopobium rimae* |
| **Firmicutes,** *s-Bacillus megaterium* |
| **Bacteroidetes,** *s-Bacteroides fragilis* |
| **Bacteroidetes,** g-Bacteroides spp. |
| **Bacteroidetes** *s-Bacteroides stercoris* |
| **Actinobacteria,** g-Bifidobacterium spp. |
| **Firmicutes,** c-Clostridia |
| **Firmicutes,** *s-Coprobacillus cateniformis* |
| **Firmicutes,** *s-Desulfitispora alkaliphila* |
| **Firmicutes,** *s-Dialister invisus* |
| **Firmicutes,** *s-Dialister invisus & s-Megasphaera micronuciformis* |
| **Firmicutes,** g-Dorea spp. |
| **Proteobacteria,** f-Enterobacteriaceae |
| **Firmicutes,** *s-Eubacterium rectale* |
| **Firmicutes,** *s-Eubacterium siraeum* |
| **Firmicutes** (various)* |
| **Firmicutes** |
| **Firmicutes,** f-Lachnospiraceae |
| **Firmicutes,** g-Lactobacillus spp. 2 |
| **Bacteroidetes*,*** *s-Parabacteroides johnsonii* |
| **Bacteroidetes,** g-Parabacteroides spp. |
| **Firmicutes,** g-Phascolarctobacterium sp. |
| **Bacteroidetes,** *s-Prevotella nigrescens* |
| **Proteobacteria,** g-Pseudomonas spp. |
| **Firmicutes,** *s-Ruminococcus gnavus* |
| **Proteobacteria,** g-Shigella spp. & g-Echerichia spp. |
| **Firmicutes,** *s-Streptococcus agalactiae* and *s-Eubacterium rectale* |
| **Firmicutes,** *s-Streptococcus salivarius ssp. thermophilus* and *s- S. sanguinis* |
| **Firmicutes,** g-Veillonella spp. |

**Additional file 1: Table S3**

Correlations (Spearman´s Rho) between Probe Signal Intensity (PSI) of fecal bacteria and fecal calprotectin and parameters reflecting disease activity and function in 150 patients with ankylosing spondylitis. All correlations with a p-value ≤0.05 are shown. A Bonferroni corrected p-value of <0.0009 was considered statistically significant (marked with *).

| **Phylum**,  c-class, o-order,  f-family, g-genus, *s-species* | Fecal  calprotectin  mg/kg | ASDAS- CRP  score | BASDAI  score | BASMI  score | BASFI  score | ESR  mm/h | CRP  mg/L |
| --- | --- | --- | --- | --- | --- | --- | --- |
| **Bacteroidetes**,  *s*-*Bacteroides pectinophilus* | -0.313*  p=0.000097 |  |  |  |  |  |  |
| **Firmicutes**,  *s-Clostridium methylpentosum* | -0.354*  p=0.000009 |  |  |  |  |  |  |
| **Actinobacteria**,  c-Actinomycetales | -0.309*  p=0.00012 |  |  |  |  |  |  |
| **Firmicutes**,  g-Streptococcus | 0.397*  p=4.98 E^-7^ |  |  |  |  |  |  |
| **Firmicutes**,  g-Streptococcus | 0.398*  p=4.67 E^-7^ |  | 0.194  p=0.018 |  |  |  |  |
| **Firmicutes**,  g-Streptococcus | 0.299*  p=0.0002 |  | 0.164  p=0.046 |  |  | 0.252  p=0.002 |  |
| **Firmicutes**, *s*-*Streptococcus sanguinis thermophilus* | 0.284*  p=0.00042 |  |  |  |  | 0.163  p=0.047 |  |
| **Firmicutes**,  *s*-*Catenibacterium mitsuokai* | 0.344*  p=0.000017 |  | 0.172  p=0.036 |  |  | 0.167  p=0.041 |  |
| **Bacteroidete**s, g-Bacteroides spp. & g-Prevotella spp. | -0.316*  p=0.000083 |  |  | -0.257  p=0.002 | -0.187  p=0.022 |  |  |
| **Firmicutes**  c-Bacilli | 0.380*  p=0.000002 | 0.200  p=0.014 | 0.216  p=0.008 |  |  | 0.195  p=0.017 |  |
| **Firmicutes**,  *s-Faecalibacterium prausnitzii* | -0.236  p=0.004 |  |  | -0.176  p=0.031 | -0.175  p=0.033 |  |  |
| **Bacteroidetes**,  g-Alistipes | -0.278  p=0.001 |  |  | -0.165  p=0.044 |  |  |  |
| **Firmicutes,**  *s-Coprobacillus cateniformis* | -0.215  p=0.008 |  |  |  |  |  |  |
| **Firmicutes,**  g-Phascolarctobacterium sp. | -0.249  p=0.002 |  |  |  |  |  |  |
| **Actinobacteria,**  c-Actinobacteria | 0.234  p=0.004 |  |  |  |  |  |  |
| **Proteobacteria**,  f-Enterobacteriacea |  |  |  |  |  | 0.229  p=0.005 |  |
| **Firmicutes**,  g-Dorea spp | 0.267  p=0.001 |  |  |  |  |  |  |
| **Actinobacteria**, g-Bifidobacterium sp. | 0.233  p=0.004 |  |  |  |  |  |  |
| **Firmicutes**,  g-Lactobacillus spp. | 0.240  p=0.003 |  |  |  | 0.172  p=0.036 |  |  |
| **Firmicutes**,  c-Clostridia | -0.232  p=0.004 |  |  |  |  |  |  |
| **Firmicutes**, *s-Ruminococcus albus & s-R. bromii* |  | -0.203  p=0.013 | -0.175  p=0.033 | -0.192  p=0.019 | -0.256  p=0.002 |  |  |
| **Proteobacteria**, g-Shigella spp. & g-Echerichia spp. |  |  |  | 0.178  p=0.029 |  | 0.174  p=0.033 |  |
| **Firmicutes**,  *s-Ruminococcus gnavus* |  |  |  |  |  |  |  |
| **Firmicutes**,  *s-Dialister invisus* |  |  |  |  |  |  |  |
| **Bacteroidetes**,  *s-Prevotella nigrescens* |  |  |  |  |  |  |  |

ASDAS=Ankylosing Spondylitis Disease Activity Score, BASDAI=Bath Ankylosing Spondylitis Disease Activity Index, BASFI=Bath Ankylosing Spondylitis Functional Index, BASMI= Bath Ankylosing Spondylitis Metrology Index, ESR= erythrocyte sedimentation rate, CRP= C- reactive protein,

**Additional file 1: Table S4**

Comparison of the fecal microbiota composition in groups of ankylosing spondylitis patients with dichotomized levels (below vs. above median value and first vs. fourth quartile) of indices of disease activity, back-mobility and function. Comparisons were also made between users vs. non-users of medication and smokers vs. non-smokers. Orthogonal partial least squares discriminant analysis (OPLS-DA) was used to define fecal microbial differences between the groups. The quality of OPLS-DA was based on the parameters R2, i.e., the goodness of fit of the model (values of ≥0.5 define good discrimination, best possible fit, R2= 1), and Q2, i.e., the goodness of prediction of the model (values of ≥0.5 define high predictive ability).

| **Y variables** | **R2** | **Q2** |
| --- | --- | --- |
| HLAB27 (positive vs negative) | 0.11 | - 0.08 |
| BASDAI (below vs. above median value) | 0.21 | -0.22 |
| BASDAI (first vs fourth quartile) | 0.47 | -0.27 |
| ASDAS-CRP (below vs. above median value) | 0.15 | -0.08 |
| ASDAS-CRP (first vs. fourth quartile) | 0.19 | 0.02 |
| BASMI (below vs. above median value) | 0.16 | -0.15 |
| BASMI (first vs. fourth quartile) | 0.25 | -0.04 |
| BASFI (below vs. above median value) | 0.152 | -0.18 |
| BASFI (first vs. fourth quartile) | 0.29 | 0.11 |
| CRP (below vs. above median value) | 0.154 | -0.101 |
| CRP (first vs. fourth quartile) | 0.25 | -0.039 |
| ESR (below vs. above median value) | 0.14 | -0.16 |
| ESR (first vs. fourth quartile) | 0.20 | -0.08 |
| NSAID (users vs. non-users) | 0.21 | 0.03 |
| TNFi (users vs. non-users) | 0.22 | 0.10 |
| DMARD (users vs. non-users) | 0.17 | 0.02 |
| Smoking (current smokers vs non-smokers) | 0.13 | -0.12 |
