# Supplementary figures and images for: A distinct gut microbiota composition in patients with ankylosing spondylitis is associated with increased levels of fecal calprotectin
Source: Arthritis Res Ther. 2019 Nov 27;21:248. doi: 10.1186/s13075-019-2018-4 (PMC6880506; doi:10.1186/s13075-019-2018-4)

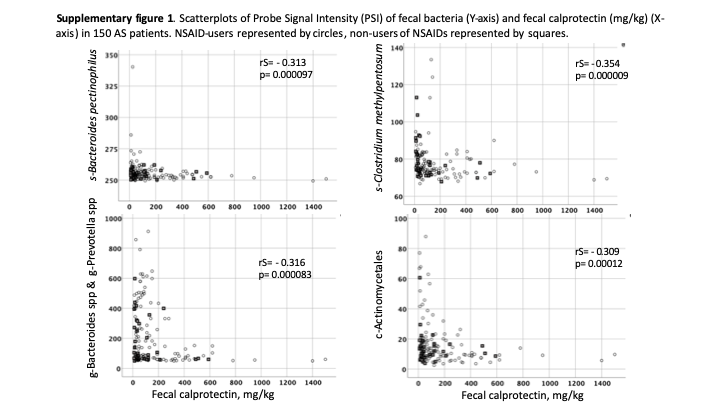

Supplement: Supplementary file 2 — Additional file 2: Figure S1. Scatterplots of Probe Signal Intensity (PSI) of fecal bacteria (Y-axis) and fecal calprotectin (mg/kg) (X-axis) in AS patients. Patients on NSAIDs are represented by circles, patients not on NSAIDs represented by squares. [file 13075_2019_2018_MOESM2_ESM.zip › Supplementary figure 1 part 1.tiff]

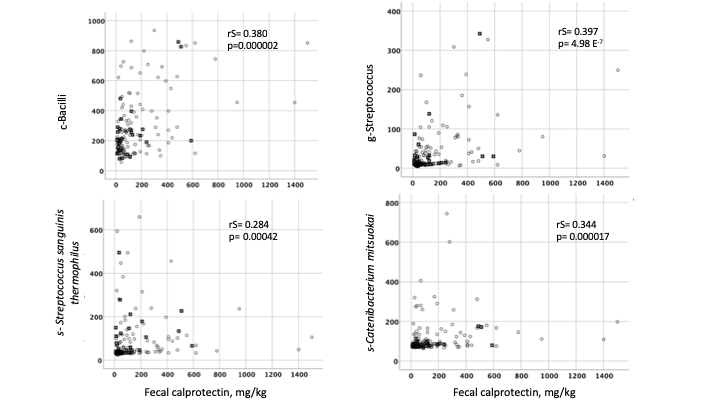

Supplement: Supplementary file 2 — Additional file 2: Figure S1. Scatterplots of Probe Signal Intensity (PSI) of fecal bacteria (Y-axis) and fecal calprotectin (mg/kg) (X-axis) in AS patients. Patients on NSAIDs are represented by circles, patients not on NSAIDs represented by squares. [file 13075_2019_2018_MOESM2_ESM.zip › Supplementary figure 1 part 2.tiff]

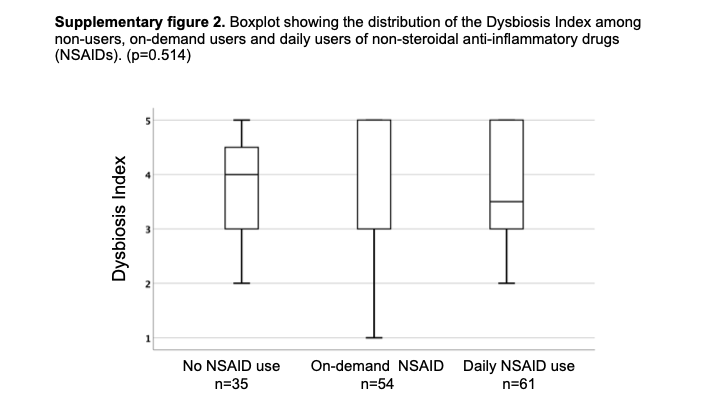

Supplement: Supplementary file 3 — Additional file 3: Figure S2. Boxplot showing the distribution of the Dysbiosis Index among non-users, on-demand users and daily users of non-steroidal anti-inflammatory drugs (NSAIDs). [file 13075_2019_2018_MOESM3_ESM.tiff]

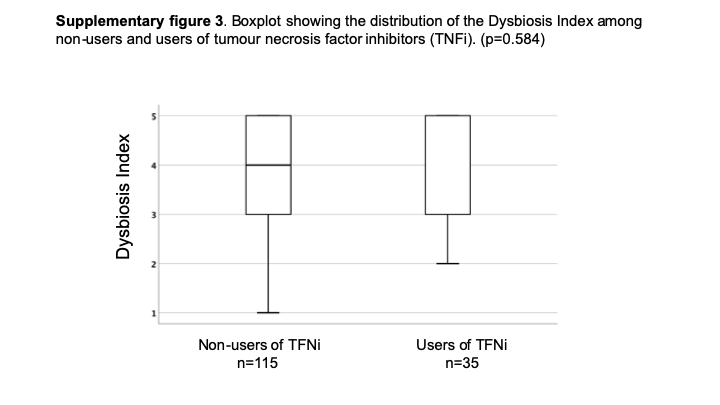

Supplement: Supplementary file 4 — Additional file 4: Figure S3. Boxplot showing the distribution of the Dysbiosis Index among non-users and users of tumour necrosis factor inhibitors (TNFi). [file 13075_2019_2018_MOESM4_ESM.tiff]
